# Fibrosis of Periprostatic Adipose Tissue: A Potential Marker of Prostate Cancer Aggressiveness

**DOI:** 10.3390/cancers18060949

**Published:** 2026-03-14

**Authors:** Yiling Jin, Jinyue Hu, Gang Wang, Yu Zhang, Zhiming Bai, Mengxing Huang, Jing Chen

**Affiliations:** 1 Department of Radiology, Affiliated Haikou Hospital of Xiangya Medical College, Central South University, Haikou 570208, China; jinyiling36@163.com (Y.J.); jinyue_hu@163.com (J.H.); 2 Department of Urology, Affiliated Haikou Hospital of Xiangya Medical College, Central South University, Haikou 570208, China; wanggang_doctor@126.com (G.W.); hkbzm59@aliyun.com (Z.B.); 3 College of Computer Science and Cyberspace Security, Hainan University, Haikou 570228, China; yuzhang2015@hainanu.edu.cn; 4 College of Information and Communication Engineering, Hainan University, Haikou 570208, China; huangmx09@163.com

**Keywords:** periprostatic adipose tissue, prostate cancer, fibrosis, radiomics

## Abstract

**Background**: Periprostatic adipose tissue (PPAT) fibrosis is a histological feature potentially linked to prostate cancer (PCa) aggressiveness, though its role is not fully understood. This study investigates the correlation between PPAT fibrosis and PCa aggressiveness and develops a radiomics model based on PPAT MRI features for non-invasive prediction. **Methods**: This retrospective study included 51 patients who underwent radical prostatectomy. PPAT samples were collected, stained with Sirius Red and quantitatively evaluated for fibrosis using 12 indices via 3D reconstruction with Imaris software. Patients were stratified by cancer aggressiveness based on Grade Groups. Radiomic features were extracted from T1-weighted MRIs of the PPAT. An XGBoost model was developed to predict aggressiveness using these features. **Results**: Significant correlations were found between multiple PPAT fibrosis indices and PCa aggressiveness (*p* < 0.05), with more aggressive tumors showing increased fiber complexity. PPAT fibrosis was also significantly associated with primary tumor location in the peripheral zone (*p* < 0.05). Conversely, PPAT volume showed no significant correlation with aggressiveness (*p* = 0.616). The radiomics model based on PPAT features achieved an AUC of 0.86 in predicting cancer aggressiveness. **Conclusions**: PPAT fibrosis is a promising marker of PCa aggressiveness, superior to PPAT volume. The significant link with tumor location provides new insights into the tumor microenvironment (TME). MRI-based radiomics of PPAT offers a potential non-invasive method for assessing fibrosis and aggressiveness, aiding in early diagnosis and personalized treatment.

## Simple Summary

This study investigated the feasibility of using periprostatic adipose tissue (PPAT) fibrosis as a potential marker of prostate cancer aggressiveness. A quantitative analysis of PPAT fibrosis was conducted on samples from 51 patients who underwent radical prostatectomy. The results revealed that more aggressive tumors were associated with increased complexity in the fibrous structure of PPAT, including an increased number of fibers, disorganized distribution and altered physical properties. This is the first study to report a significant correlation between the degree of PPAT fibrosis and the primary tumor location in prostate cancer. This study further validated the feasibility of evaluating PPAT fibrosis via MRI-based radiomic features, thus suggesting its potential as a noninvasive method to facilitate early diagnosis and personalized treatment of prostate cancer.

## 1. Introduction

With the increasing global trend of population aging, prostate cancer has become one of the most common malignancies among men [1]. This is particularly true in developing countries, where most cases of prostate cancer are diagnosed at the middle-to-late stages, resulting in a generally poor prognosis [2]. In recent years, periprostatic adipose tissue (PPAT) has attracted attention because of its potential association with the aggressiveness of prostate cancer [3,4,5]. Within the tumor microenvironment (TME), PPAT fibrosis has emerged as a key histological feature [6,7,8]. However, the functional role of PPAT in prostate cancer remains controversial. Previous studies [9,10] have suggested that PPAT may exert a protective effect in early-stage prostate cancer, as an intact adipose microenvironment may inhibit initial tumorigenesis and local proliferation. In contrast, a growing consensus links pathological remodeling of PPAT to accelerated aggressiveness of prostate cancer [11]. Therefore, the mechanistic connection between PPAT fibrosis and prostate cancer aggressiveness has not been fully elucidated; the relationship among fibrosis, tumor location and conventional clinical prognostic markers remains insufficiently characterized. The translational potential of PPAT fibrosis as a non-invasive predictive marker remains to be validated. Current evaluation of PPAT fibrosis mainly relies on invasive histological analysis of surgical specimens. Addressing these knowledge gaps is critical for clarifying the paradoxical role of PPAT in prostate cancer and identifying novel non-invasive biomarkers for early diagnosis and risk stratification. Accordingly, this study aims to systematically investigate the correlation between quantitative indices of PPAT fibrosis and prostate cancer aggressiveness, characterize the association between PPAT fibrosis and the location of primary tumors within the prostate gland and explore the feasibility of non-invasive assessment of PPAT fibrosis and prediction of prostate cancer aggressiveness using MRI-based radiomic features.

## 2. Materials and Methods

### 2.1. Study Subjects

This retrospective, single-center, cross-sectional study collected data from prostate cancer patients who underwent robotic-assisted laparoscopic radical prostatectomy (RALP) at Xiangya Hospital, Central South University, Haikou, from October 2020 to May 2023. Ethical approval was obtained from the Ethics Review Committee of Xiangya Hospital, Central South University, Haikou (Protocol Number: 2023-(lunshen)-198).

**Figure 1:**
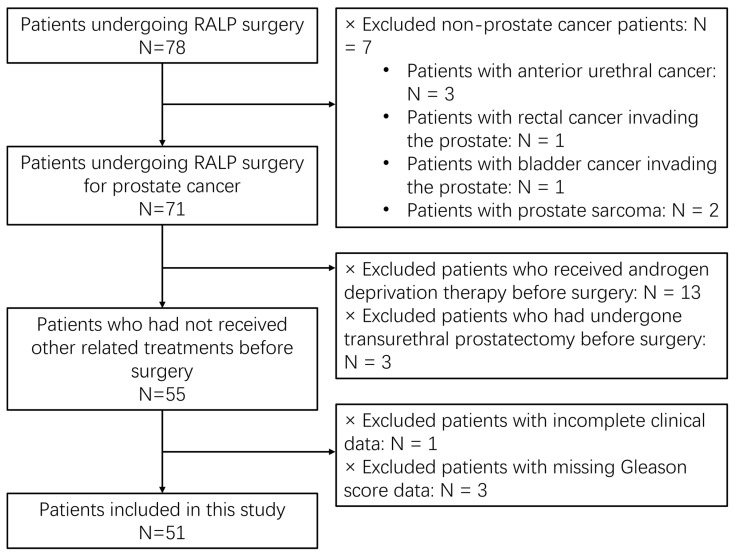
Selection of study subjects.

The inclusion criteria were as follows: Patients who underwent radical prostatectomy for prostate cancer; Patients who had undergone pelvic MRI prior to prostate biopsy; Patients whose PPAT samples were obtained during radical prostatectomy. The exclusion criteria included the following: Patients with non-prostatic adenocarcinoma; Patients with incomplete basic clinical data; Patients with incomplete pathological data, such as missing Gleason scores (GSs); Patients who had undergone transurethral resection of the prostate (TURP); Patients who had received endocrine therapy, radiotherapy, or other nonsurgical treatments prior to surgery; Patients with incomplete or poor-quality pelvic MR images. A total of 51 patients met the inclusion criteria for this study, as shown in Figure 1. The baseline characteristics of these patients are summarized in Table 1. All patients underwent clinical evaluation, PSA testing, pelvic MRI and RALP. Additionally, two radiologists with 6–8 years of experience in diagnostic imaging independently reviewed all multiparametric MRI (mpMRI) scans. Pathological evaluation, including an assessment of the Gleason score, was performed by pathologists. To ensure objectivity and accuracy, the radiologists were blinded to the pathological results and the pathologists were blinded to the mpMRI findings.

### 2.2. Cohort Stratification

To minimize bias and ensure balance across groups, the included cases were stratified based on aggressiveness. Using the prostate cancer Grade Group (GG) system [12], which indicates varying mortality risks for patients in different grade groups [13], the cases were divided into the following categories: The cohort comprised 14 patients (27.45%) in the low-aggressiveness group, 19 patients (37.25%) in the moderate-aggressiveness group and 18 patients (35.29%) in the high-aggressiveness group. The detailed criteria for stratification are shown in Table 2.

### 2.3. Quantitative Acquisition of PPAT Fibrosis Data

**Figure 2:**
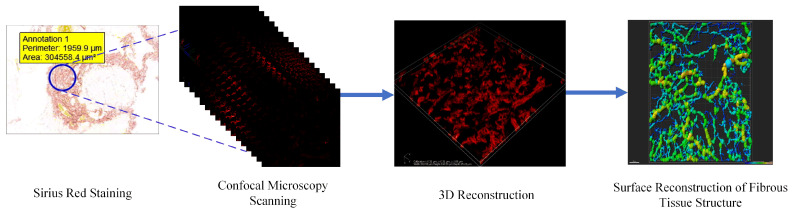
Quantitative acquisition of PPAT fibrosis data. This figure illustrates the workflow for obtaining quantitative data on PPAT fibrosis: 1. **Tissue Collection**: PPAT samples were collected during robotic-assisted laparoscopic radical prostatectomy (RALP). 2. **Histological Processing**: Specimens were embedded in paraffin and sectioned for analysis. 3. **Staining and Imaging**: Sections were stained with Sirius Red and imaged via a Nikon laser confocal microscope at 40× magnification, with an excitation wavelength of 560 nm and fluorescence emission signals above 580 nm. 4. **Data Reconstruction**: The images were processed with the Filament Tracer module in Imaris software (version 9.0.1) for 3D collagen fiber reconstruction. 5. **Data Standardization**: Quantitative data were standardized for subsequent analysis. **Color explanation**: The red color represents the Sirius Red-stained collagen fibers of PPAT under fluorescence imaging and the background color corresponds to the non-fibrotic tissue components of PPAT with no specific fluorescence signal. The cool to warm tones of the reconstructed PPAT fibers indicate an increase in fiber diameter from thin to thick.

The RALP procedures were performed by urologists at Xiangya Hospital, Central South University, Haikou, who were certified with Da Vinci surgical system training. During surgery, PPAT samples were collected and subsequently processed into paraffin-embedded samples by pathologists at the same institution. Following preparation, the PPAT sections were stained with Sirius Red and imaged with a Nikon laser confocal microscope. The imaging parameters included a magnification of 40×, an excitation wavelength of 560 nm and fluorescence emission signals collected at wavelengths above 580 nm. The images were saved in ND2 format. The three-dimensional reconstruction of the collagen fiber network was performed using the Filament Tracer module in Imaris software (version 9.0.1). This automated module identifies and tracks filamentous structures, thereby quantifying 12 distinct fibrotic indices to provide a quantitative assessment of the degree of fibrosis in the PPAT. After surface reconstruction of the tissue in Imaris, data were generated and standardized for further analysis. The entire process is illustrated in Figure 2.

Fibrosis Evaluation Indicators (i.e., the Fibrosis Index (FI)):

1. Filament Length: Total length of fiber structures, measured in μm.
2. Filament Area: Total surface area covered by fiber structures, measured in μm^2^.
3. Filament Volume: Total volume of fiber structures, measured in μm^3^.
4. Dendrite Mean Diameter: Average diameter of fiber branches after 3D reconstruction, measured in μm.
5. Dendrite Straightness: Comparison of the actual path of fiber branches to the direct line between their endpoints.
6. Dendrite Branching Angle: Angle between fiber branches and the main axis, measured in degrees (°).
7. Dendrite Orientation Angle: Overall direction of fiber branches, reflecting alignment within the tissue, measured in degrees (°).
8. Filament No. Dendrite Branch Pts: Number of branch points in the fiber structures.
9. Filament No. Dendrite Branches: Total count of fiber branches.
10. Filament No. Dendrite Segments: Number of individual segments between branch points within the fiber structure.
11. Filament No. Dendrite Terminal Pts: Total number of terminal points in the fiber structures.
12. Filament No. Sholl Intersections: Number of intersections between fiber branches and concentric circles of increasing radii from the fiber’s starting point, as determined by Sholl analysis.

These indicators comprehensively quantify the overall and local characteristics of PPAT fibrosis, including the structural arrangement and physical property changes. Metrics such as the filament length, area and volume reflect the overall structural status of the fibers. The mean diameter indicates the thickness of the fibers. The straightness, branching angle and orientation angle affect the alignment and arrangement of the fibers. The remaining indicators, including branch points, segments, terminal points and Sholl intersections, provide insights into changes in physical properties, thus enabling an integrated assessment of structural complexity. Of note, these mechanical characteristics represent indirect surrogate markers rather than direct quantitative measurements.

### 2.4. MR Imaging Selection and Processing

#### 2.4.1. Selection of MR Images

**Figure 3:**
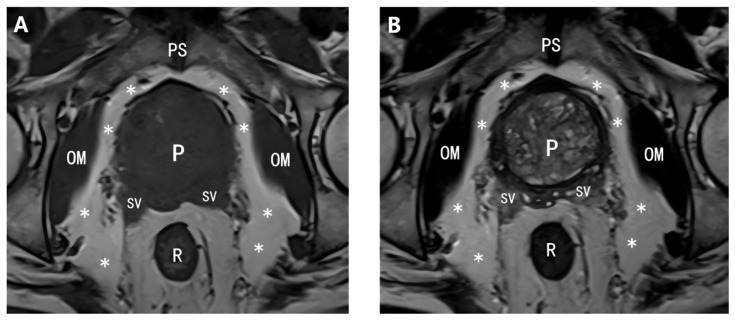
Prostate MR images. (**A**) Axial T1-weighted MR image of the prostate. (**B**) Axial T2-weighted MR image of the prostate. The asterisk (*) indicates periprostatic adipose tissue (PPAT). **Abbreviations**: **PS**: Pubic symphysis; **OM**: Obturator internus muscle; **P**: Prostate; **SV**: Seminal vesicle; **R**: Rectum.

MR images were acquired via two MRI scanners at Xiangya Hospital, Central South University, Haikou: the MAGNETOM Skyra 3T superconducting MRI system (Siemens, Erlangen, Germany) and the 3.0 T SIGNA HDX MRI scanner (GE, Chicago, USA). T2-weighted imaging (T2WI) is the primary sequence for evaluating the anatomical structure of the prostate gland [14,15]. Axial views are considered the optimal angle for observing the prostate on MR images. Therefore, we selected axial T2WI sequences to annotate the primary lesion of prostate cancer in the included patients and recorded the maximum diameter and location (peripheral zone, central zone). For PPAT annotation, T1-weighted imaging (T1WI) was used. As shown in Figure 3, in our dataset, axial T1WI images provided better delineation of the prostate gland and PPAT contours than axial T2WI images. Thus, T1WI images were deemed more suitable for distinguishing these two structures and served as the primary data source. This approach aligns with the findings of Zhai et al. and Liu et al. [16,17].

All MR images were in DICOM format. Using Python (version 3.9.7), all DICOM files were converted into the NIfTI format to increase the data processing efficiency and cross-platform compatibility. Furthermore, to minimize the impact of data instability on the study and improve the performance of machine learning methods, all images were preprocessed. The preprocessing steps included resampling, resizing and normalization [18].

#### 2.4.2. Delineation of PPAT Boundaries and Regions of Interest (ROIs) with Volume Calculation

**Figure 4:**
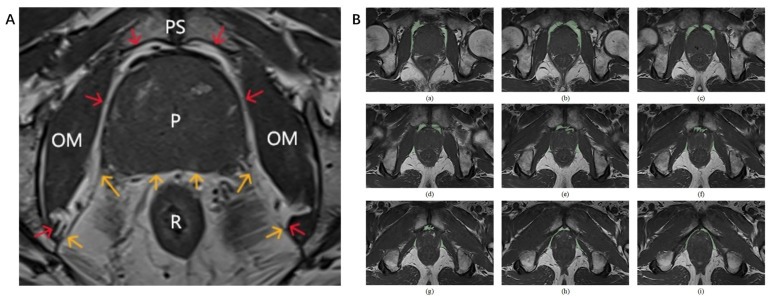
Delineation of PPAT. (**A**): The PPAT boundaries are indicated by the area between the red and yellow arrows and the prostate contour. The yellow arrow points to the boundary between the perirectal fat and Denonvilliers’ fascia. (**B**): Panels (**a**–**i**) illustrate the step-by-step delineation of different layers from the base to the apex of the prostate. **Abbreviations**: **PS**: Pubic symphysis; **OM**: Obturator internus muscle; **P**: Prostate; **R**: Rectum.

The boundaries of the PPAT were defined as follows [19,20,21]: anteriorly by the pubic symphysis, laterally by the obturator internus muscles, posteriorly by Denonvilliers’ fascia (excluding mesorectal fat), superiorly by the bladder and inferiorly by the urethral sphincter. This definition also includes the anterior venous plexus and retropubic fat, as shown in Figure 4A.

In this study, the drawing tools provided in the Segment Editor module of 3D Slicer software (version 5.2.2) were used to manually delineate PPATs on T1WI images from the base to the apex of the prostate (Figure 4B). Similarly, tumors were segmented layer by layer on T2-weighted MR images. All annotations were subsequently reviewed by radiologists with 6–8 years of experience in diagnostic imaging. Given that most recent studies used the PPAT thickness or volume as potential markers of prostate cancer aggressiveness [22,23], we measured the PPAT volume and compared its predictive value with that of PPAT fibrosis for prostate cancer aggressiveness. The PPAT volume data for 51 patients were calculated via the segment statistics function in the Models module of 3D Slicer software. The unit of measurement was cubic millimeters (mm^3^).

#### 2.4.3. Segmentation Consistency Validation

Segmentation and radiomic feature extraction of the PPAT from T1WI images are critical steps requiring rigorous validation. Inter-observer and intra-observer agreement were assessed in this study. Two board-certified radiologists with 6–8 years of genitourinary imaging experience independently delineated PPAT in 20 randomly selected cases. Inter-observer agreement was quantified using the Dice similarity coefficient and Hausdorff distance, while intra-observer reproducibility was evaluated by one reader after a four-week interval using the Dice coefficient. Radiomic features with Intraclass Correlation Coefficient (ICC) > 0.75 in both tests were retained as robust features. This validation ensures reliable region-of-interest definitions and enhances the credibility of imaging-based predictive models.

#### 2.4.4. Extraction of Radiomic Features

Radiomic feature extraction was performed via the open-source Python library PyRadiomics (version 1.2.0) [24]. A total of 1409 radiomic features were extracted for each patient, including the following: first-order statistical features, shape and size features, texture features based on texture matrices and filter-based features. Detailed formulas for each type of radiomic feature are available on the PyRadiomics website “https://pyradiomics.readthedocs.io/en/latest/ (accessed on 27 December 2025)”. To ensure consistency in feature dimensions during model training, the raw radiomic feature data were standardized via the StandardScaler function, which adjusted the mean of each feature to 0 and the standard deviation to 1. Ridge regression was subsequently employed to calculate the radiomics score (rad-score). All data processing and model training were performed via the PyCharm integrated development environment (version 2023.3.4), with Python “https://www.python.org/ (accessed on 13 January 2026)” used as the programming language.

#### 2.4.5. Development of a Prostate Cancer Aggressiveness Prediction Model

**Figure 5:**
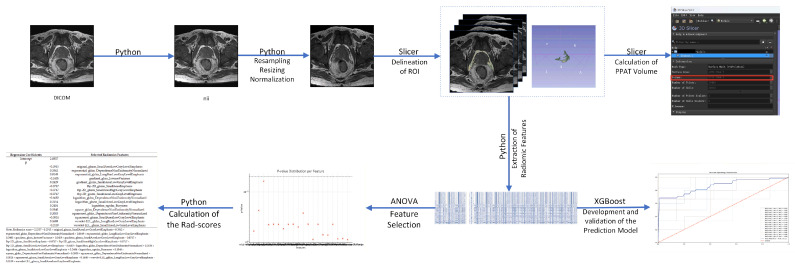
The entire radiomics analysis workflow. **ANOVA**: Analysis of variance. The red square represents the data of PPAT volume. The letters correspond to the following anatomical planes: P = posterior, A = anterior, L = left, R = right and S = Superior.

The dataset of 51 cases was randomly shuffled to ensure random distribution. Given the small sample size, we employed the XGBoost algorithm as the framework to build the model, minimizing the risk of overfitting. XGBoost was chosen for its ability to handle small datasets efficiently, with built-in regularization to prevent overfitting and faster performance through parallel computation [25,26]. A fivefold cross-validation strategy was applied, with the dataset divided into a training set (41 cases) and a validation set (10 cases). The training set was used to construct the model, while the validation set was used to assess its generalization ability. Additionally, hyperparameter tuning was conducted to further optimize model performance. The objective was to identify the best combination of parameters to improve accuracy and performance, thus ensuring that the model’s precision and overall effectiveness were maximized. Figure 5 illustrates the complete radiomic analysis workflow.

### 2.5. Statistical Analysis

In this study, continuous variables are expressed as the means ± standard deviations (x ± s), whereas categorical variables are presented as frequencies. Statistical analysis was performed using SPSS software (version 26.0) and Python. Analysis of variance (ANOVA) was used for comparisons among more than three groups of continuous variables. A *t*-test was employed for two-group comparisons. Pearson’s correlation analysis was used to evaluate linear relationships between continuous variables. Spearman’s correlation analysis was used for ordinal data correlations. Ridge regression was applied for multivariate analysis. Statistical significance was defined as *p* < 0.05 and *p* < 0.01 was considered highly significant.

## 3. Results

### 3.1. Correlation Between PPAT Fibrosis and Prostate Cancer Aggressiveness

**Figure 6:**
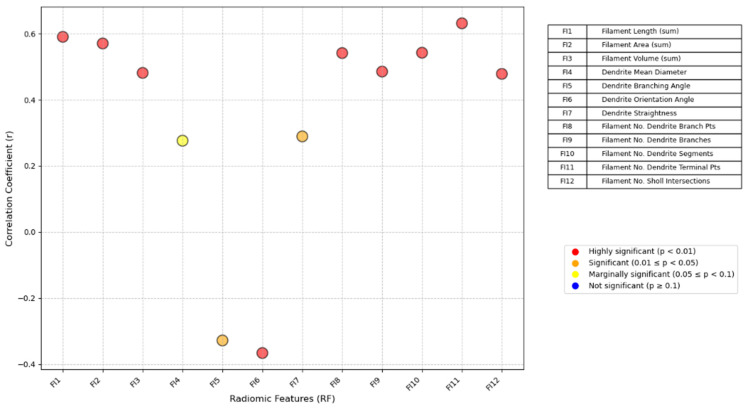
Correlation between PPAT fibrosis and prostate cancer aggressiveness.

Spearman’s correlation analysis revealed a statistically significant relationship between PPAT fibrosis indices and prostate cancer aggressiveness (Figure 6). The following fibrosis indices were significantly correlated with aggressiveness (*p* < 0.01): fiber length, fiber area, fiber volume, branch orientation angle, number of branch points, number of branches, number of independent segments, number of terminal points and number of Sholl intersections. Additionally, the mean branch diameter, branching angle and branch straightness were correlated with aggressiveness (*p* < 0.05) (Supplementary Table S1). These results indicate that as prostate cancer aggressiveness increased: the area, length and volume of the PPAT fibers increased; the fiber diameters became larger; the arrangement and distribution of fibers became more disordered; the structural complexity of the fibers increased. In contrast, the PPAT volume was not significantly correlated with prostate cancer aggressiveness (*p* = 0.616). These findings suggest that, compared with PPAT volume, PPAT fibrosis may serve as a superior potential marker of prostate cancer aggressiveness.

Additionally, we analyzed the correlation between the PPAT volume and the PPAT fibrosis indices (Supplementary Figure S1). The results revealed statistically significant correlations for the following indices: fiber Length (*p* = 0.005), fiber Area (*p* = 0.002), fiber Volume (*p* = 0.010) and number of Terminal Points (*p* = 0.016). However, no statistically significant correlations were detected between the PPAT volume and the remaining fibrosis indices. Previous studies [27,28,29] have explored the associations between the PPAT volume and body mass index (BMI), utilizing the PPAT volume as a predictive marker for assessing the malignancy of prostate cancer. Our findings suggest that PPAT fibrosis indices may serve as superior potential markers of prostate cancer aggressiveness compared with the PPAT volume.

### 3.2. Correlation Between PPAT Fibrosis and Prostate Tumor Location

**Figure 7:**
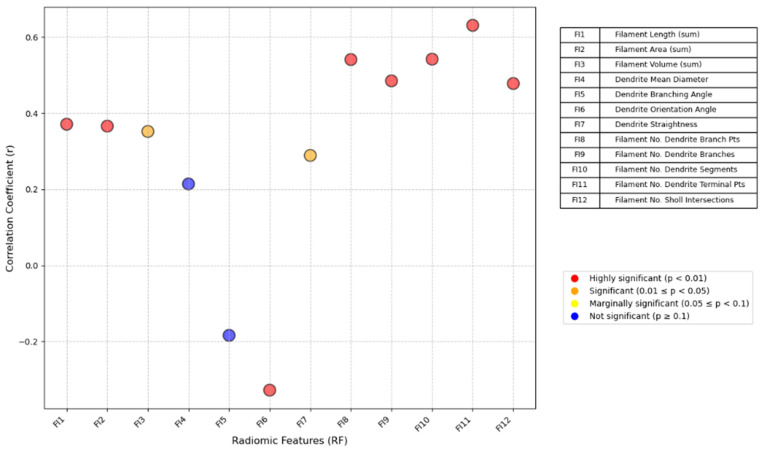
Correlations between PPAT fibrosis and prostate tumor location.

**Figure 8:**
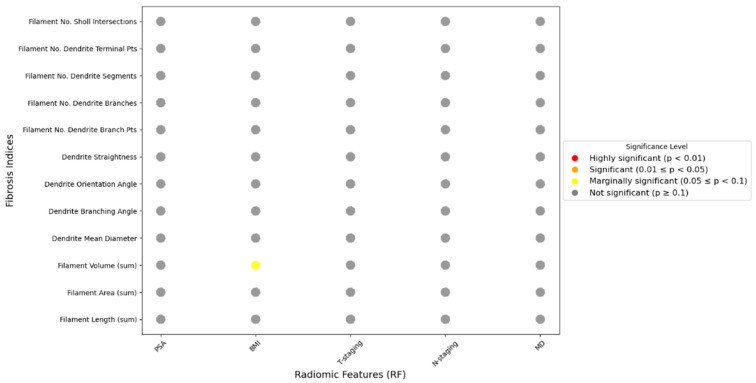
Correlations between PPAT fibrosis and PSA levels, BMI, T Staging, N Staging and Maximum Diameter of the Primary Lesion (MD).

Spearman’s correlation analysis revealed a statistically significant relationship between PPAT fibrosis indices and the location of the primary lesion in prostate cancer patients (Figure 7). Prior to this analysis, we evaluated potential confounding variables, including tumor grade group and maximum diameter of the primary lesion between PZ and central zone tumors, with no significant differences being identified in either tumor grade (*p* = 0.382) or maximum diameter (*p* = 0.415) between the two subgroups. The following fibrosis indices were strongly correlated with tumor location: fiber length (r = 0.371, *p* < 0.01), fiber area (r = 0.366, *p* < 0.01) and number of terminal points (r = 0.395, *p* < 0.01). Additionally, the following indices were significantly correlated with tumor location: fiber volume (r = 0.352, *p* < 0.05), branch orientation angle (r = −0.315, *p* < 0.05), number of branch points (r = 0.35, *p* < 0.05), number of branches (r = 0.28, *p* < 0.05), number of independent segments (r = 0.35, *p* < 0.05) and number of Sholl intersections (r = 0.278, *p* < 0.05) (Supplementary Table S2). These findings indicate that patients with primary tumors located in the peripheral zone of the prostate tend to exhibit increased degrees of PPAT fibrosis. This is characterized by an increased number of fibers, greater stiffness, reduced elasticity and more disorganized fiber arrangement. In addition, we assessed the correlation between the maximum diameter of the primary lesion and PPAT fibrosis, but found no statistically significant results, as shown in Figure 8. These findings suggest that the characteristics of PPAT fibrosis are more influenced by the location than the size of the tumor. Tumors located in the peripheral zone may be more likely to induce fibrotic responses in the surrounding adipose tissue.

These results highlight that tumors in the peripheral zone are associated with more severe PPAT fibrosis, characterized by increased fiber quantity, greater stiffness, reduced elasticity and more disorganized arrangements. Furthermore, the correlation between extraprostatic extension (ECE) status and the PPAT fibrosis index was evaluated in the peripheral zone subgroup. Among the 51 patients, T-stage data were available for 40 cases, with the following distribution: ≤T2 stage (*n* = 27) and ≥T3 stage (*n* = 13). Based on pathological assessment of radical prostatectomy specimens, 8 out of 24 patients (33.3%) with peripheral zone tumors were found to have ECE. We also assessed the correlation between PSA levels, BMI and PPAT fibrosis in the included patients (Supplementary Table S3); analyzed differences in PPAT fibrosis severity according to T stage and N stage (non-metastatic: N0; metastatic: ≥N1). However, as shown in Figure 8, although an increasing trend in the fibrosis index was observed in ECE-positive cases, these differences did not reach statistical significance. These findings suggest that, compared with PSA levels, BMI and TNM staging, the progression of prostate cancer aggressiveness may be more prominently reflected in the early increase in the degree of PPAT fibrosis.

The results revealed no statistically significant correlations, indicating that PPAT fibrosis is less influenced by these factors. Instead, the degree of PPAT fibrosis may serve as a more sensitive early marker of prostate cancer aggressiveness than these conventional parameters.

### 3.3. Validation of the Relationships Between PPAT Radiomic Features and the Degree of Fibrosis

Given that PPAT samples are obtained primarily through invasive radical prostatectomy, the current evaluation of PPAT fibrosis relies heavily on histological analysis. To explore the possibility of using noninvasive imaging data to assess PPAT fibrosis, we attempted to delineate PPATs on T1WI images of the included cases, extract radiomic features and validate their correlation with PPAT fibrosis indices. From the T1WI images of each patient, a total of 1409 radiomic features were extracted. After applying variance analysis to filter out features with no significant differences between groups, 16 features were ultimately identified as having statistically significant differences across groups with varying levels of risk (*p* < 0.05), as shown in Table 3. These significant features encompass a wide range of metrics derived from different image processing techniques, including but not limited to the following: original image analysis, exponential transformations, gradient transformations and local binary patterns (LBP). These results suggest that radiomic features may provide meaningful, noninvasive insights into the degree of PPAT fibrosis, potentially serving as valuable tools for clinical evaluation.

This model demonstrates the contribution of each radiomic feature (Table 4) to the rad score, which serves as a composite measure for predicting PPAT fibrosis and its association with prostate cancer aggressiveness. According to the results of Pearson’s correlation analysis, there was a statistically significant positive correlation between the radiomics score of the PPAT and the mean diameter of the fiber branches in prostate cancer patients (*p* = 0.042). This finding indicates that as the radiomics score increases, the fiber diameter becomes thicker, thus suggesting that the fiber structure may become rougher. Additionally, we attempted to develop a prostate cancer aggressiveness prediction model using PPAT radiomic features. The evaluation of the validation set demonstrated good performance of the predictive model, with an area under the curve (AUC) of 0.86, indicating high discriminatory ability. The model achieved an accuracy of 0.66, a recall of 0.72 and a precision of 0.74. The effectiveness and reliability of the predictive model in assessing prostate cancer aggressiveness highlight that PPAT radiomic features can partially reflect the cancer aggressiveness of prostate cancer patients. These findings support the potential utility of these features in noninvasive diagnostic applications.

## 4. Discussion

This study preliminarily validated the correlation between PPAT fibrosis and prostate cancer aggressiveness. For the first time, a significant association between fibrosis and peripheral zone tumors was demonstrated. Additionally, we propose a method for quantifying fibrosis from multiple perspectives, including fiber quantity, diameter, stiffness and elasticity. We also explored the feasibility of using noninvasive MRI-derived T1WI data combined with radiomic analysis to evaluate the degree of PPAT fibrosis. This study aimed to provide new insights into the role of the PPAT in prostate cancer progression and to offer novel approaches for the early diagnosis and treatment of prostate cancer.

Compared with previous studies [30,31], our findings further confirmed that PPAT fibrosis, as part of the TME, plays a regulatory role in prostate cancer cell behavior. The quantitative histological indices of PPAT fibrosis are highly consistent with the core biological characteristics of pathological fibrosis. Increases in filament length, area and volume reflect enhanced collagen deposition, a typical feature of fibrotic tissue remodeling driven by excessive extracellular matrix (ECM) synthesis and deposition. Meanwhile, elevated numbers of dendritic branch points, segments and endpoints correspond to the disorganized and hyperbranched collagen fiber architecture characteristic of pathological fibrosis, indicating the loss of oriented fiber alignment in PPAT. This deviation from normal physiological tissue architecture disrupts the physiological function of adipose tissue and creates a permissive microenvironment for prostate cancer progression, a mechanistic rationale for the superior association of PPAT fibrosis with tumor aggressiveness: unlike PPAT volume, which merely represents a structural morphological parameter of periprostatic adipose tissue without reflecting functional or pathological remodeling, PPAT fibrosis indices quantify the pathological structural and mechanical alterations of the TME, which are directly linked to the biological processes of tumor cell invasion, migration and metastatic potential, the key hallmarks of cancer aggressiveness. In the early stages of increased prostate cancer aggressiveness, the PPAT may already exhibit structural complexity, including increased fiber quantity and altered distribution patterns. This study also revealed a significant correlation between the degree of PPAT fibrosis and the location of the primary tumor, suggesting that tumors in the peripheral zone may be more likely to induce a fibrotic response in the surrounding adipose tissue. These findings highlight the role of PPAT fibrosis in remodeling the TME. An increase in PPAT fibrosis may serve as an early biomarker of increased prostate cancer aggressiveness. These findings support the hypothesis that tumors remodel surrounding tissues through the TME [32,33] and indicate that PPAT fibrosis could be a more sensitive biomarker than the PPAT volume for the early diagnosis of prostate cancer, thus facilitating early detection and timely intervention.

Although our findings indicate that increased fibrosis in PPAT is associated with tumor aggressiveness, Shao et al. [10] reported that PPAT may exert anti-tumor effects in the early stage of disease, likely mediated by adipokines that suppress cell proliferation or promote apoptosis. In contrast, as the disease progresses, the TME undergoes extensive remodeling, leading to hypoxia-induced alterations that drive adipose tissue fibrosis [34,35]. This fibrotic transition is characterized by increased collagen deposition and tissue stiffening, creating a permissive niche for cancer cell invasion and metastasis. Our study provides evidence supporting the latter hypothesis and helps reconcile these conflicting observations. It suggests that the switch of PPAT from a protective to a tumor-promoting role may be marked by the onset of fibrosis, which could represent a critical turning point in disease progression. Future studies should focus on elucidating the molecular triggers that initiate this fibrotic transition to better understand the shift between these opposing functions. Prostate cancer is highly heterogeneous and frequently exhibits multifocal growth accompanied by persistent chronic inflammation, which leads to local tissue injury and anatomical structural destruction, ultimately promoting lymph node (LN) invasion. Although the present study did not directly measure specific blood inflammatory markers in the cohort, further investigation into the correlation between inflammatory markers and PPAT fibrosis is critical for elucidating the pathophysiological mechanisms underlying the TME. Elevated systemic inflammatory indices generally indicate the establishment of a pro-inflammatory TME, which can further upregulate the expression of pro-fibrotic cytokines such as transforming growth factor-β (TGF-β) and interleukin-6 (IL-6), thereby accelerating the fibrotic process [36]. These findings provide novel insights into the mechanisms underlying periprostatic adipose fibrosis. Future studies should integrate multi-dimensional analyses of systemic inflammatory markers with quantitative histological fibrosis indices to more precisely delineate the causal relationships and molecular pathways linking local inflammation, PPAT fibrosis and prostate cancer aggressiveness.

We further demonstrated a significant association between the radiomic signature of periprostatic adipose tissue fibrosis and well-established pathological prognosticators, including higher Gleason scores and advanced pathological T-stages. These findings collectively suggest that this imaging biomarker holds considerable promise for the noninvasive risk stratification of patients based on disease aggressiveness. Furthermore, these results also provide a novel direction for future research, specifically to explore the relationship between radiologically quantified fibrosis and the tissue expression of critical profibrotic and epithelial–mesenchymal transition (EMT)-related proteins in the periprostatic adipose microenvironment.

## 5. Limitations

This study has several important limitations that must be acknowledged. First and foremost, the radiomic component of this study should currently be regarded as promising proof-of-concept work. The model was developed and validated using a limited single-center dataset of only 51 patients, with 41 for training and 10 for validation. Although the XGBoost model achieved an AUC of 0.86, no confidence interval was reported due to the small validation cohort. Importantly, external validation was not performed. Accordingly, the reported model performance should be considered preliminary. The model stability and generalizability to broader independent populations remain to be confirmed, representing a major limitation for clinical translation. Secondarily, to mitigate the risk of false-positive results (Type I error), analysis of variance was applied to filter non-significant radiomic features prior to model construction. However, formal multiple comparison correction methods, such as false discovery rate (FDR) adjustment, were not used for correlation analyses of fibrosis indices. Therefore, the significant correlations identified should be interpreted cautiously. Future work should focus on expanding the cohort size, conducting strict multiple test correction and external validation. Furthermore, we acknowledge that the analysis did not fully control for all potential confounding factors, such as precise tumor volume, extracapsular extension status, or detailed sub-regional stratification within the peripheral zone. While the biological mechanisms underlying this location-specific association remain speculative, they may be related to the anatomical proximity of the prostatic peripheral zone capsule to the PPAT. Future studies are warranted to further investigate the molecular mechanisms driving this location-specific fibrotic response.

## 6. Conclusions

Changes in the local TME of prostate cancer may exacerbate the degree of PPAT fibrosis, which, in turn, promotes tumor cell invasion and metastasis. This aspect has been underexplored in previous studies. The findings of this study contribute to a deeper understanding of the role of the prostate cancer microenvironment in tumor progression, thus providing new perspectives on the role of PPAT in prostate cancer development. This work aims to offer novel insights for the diagnosis and treatment of prostate cancer and provide a scientific basis for personalized treatment plans for prostate cancer patients.

## Figures and Tables

**Table 1:** Baseline characteristics of the included patients.

Items	Mean	Minimum	Maximum
**General Information**			
Age (years)	70	55	82
BMI (kg/m^2^)	22.58	17.11	29.73
Initial tPSA (ng/mL)	26.42	4.18	146
Gleason Score	7	6	9
TNM Staging	/	T1aN0M0	T4N1M0
**PPAT Parameters**			
PPAT Volume (mm^3^)	8213.16	1971.87	59,017.41
PPAT Indices			
Filament Length (μm)	1014.73	70.69	11,817.10
Filament Area (μm^2^)	36,111.84	1640.71	347,044.00
Filament Volume (μm^3^)	116,382.12	3109.51	1,100,130.00
Dendrite Mean Diameter (μm)	8.76	3.72	20.46
Dendrite Branching Angle (°)	47.55	40.94	59.21
Dendrite Orientation Angle (°)	−0.54	−14.59	30.72
Dendrite Straightness	0.90	0.84	0.99
Filament No. Dendrite Branch Pts	56.03	2.56	505.00
Filament No. Dendrite Branches	17.33	2.36	92.71
Filament No. Dendrite Segments	103.05	5.46	928.00
Filament No. Dendrite Terminal Pts	22.11	3.22	337.00
Filament No. Sholl Intersections	15.11	3.04	52.63
**Imaging Parameters**			
Maximum Diameter of Primary Lesion (mm)	12.94	3.98	37.39
Tumor Location	/	Central Area (27/51)	PZ (24/51)

Note: The central area includes both the central and transitional zones. TNM staging data were missing for 11 patients. The bolded text in the table is used to highlight the major parameter categories for clear classification and reading.

**Table 2:** Criteria for aggressiveness stratification.

Grade Group	Gleason Score	Gleason Pattern	Aggressiveness Classification
1	≤6	≤3 + 3	Low
2	7	3 + 4	Moderate
3	7	4 + 3	High
4	8	4 + 4, 3 + 5, 5 + 3	High
5	9 or 10	4 + 5, 5 + 4, 5 + 5	High

**Table 3:** Correlations between the PPAT volume and PPAT fibrosis indices.

Radiomic Features	*p* Value
original_glszm_SmallAreaLowGrayLevelEmphasis	0.033
exponential_gldm_DependenceNonUniformityNormalized	0.019
exponential_glrlm_LongRunLowGrayLevelEmphasis	0.049
gradient_glcm_InverseVariance	0.049
gradient_glszm_SmallAreaLowGrayLevelEmphasis	0.039
lbp-2D_glszm_SmallAreaEmphasis	0.043
lbp-2D_glszm_SmallAreaHighGrayLevelEmphasis	0.043
lbp-2D_glszm_SmallAreaLowGrayLevelEmphasis	0.043
logarithm_gldm_DependenceNonUniformityNormalized	0.022
logarithm_glszm_SmallAreaLowGrayLevelEmphasis	0.032
logarithm_ngtdm_Busyness	0.023
square_gldm_DependenceNonUniformityNormalized	0.029

Note: This table shows the significant radiomic features associated with PPAT fibrosis. These features have the potential to serve as noninvasive indicators for evaluating PPAT fibrosis and its relationship with tumor aggressiveness.

**Table 4:** Radiomic features derived from ridge regression.

Regression Coefficients		Selected Radiomics Features
Intercept	2.0357	
β		
	−0.2915	original_glszm_SmallAreaLowGrayLevelEmphasis
	0.3562	exponential_gldm_DependenceNonUniformityNormalized
	0.0149	exponential_glrlm_LongRunLowGrayLevelEmphasis
	−0.2405	gradient_glcm_InverseVariance
	0.2429	gradient_glszm_SmallAreaLowGrayLevelEmphasis
	−0.0717	lbp-2D_glszm_SmallAreaEmphasis
	−0.0717	lbp-2D_glszm_SmallAreaHighGrayLevelEmphasis
	−0.0717	lbp-2D_glszm_SmallAreaLowGrayLevelEmphasis
	−0.6683	logarithm_gldm_DependenceNonUniformityNormalized
	0.3154	logarithm_glszm_SmallAreaLowGrayLevelEmphasis
	0.2404	logarithm_ngtdm_Busyness
	0.1940	square_gldm_DependenceNonUniformityNormalized
	0.2003	squareroot_gldm_DependenceNonUniformityNormalized
	−0.0024	squareroot_glszm_SmallAreaLowGrayLevelEmphasis
	0.1688	wavelet-LLL_glrlm_LongRunLowGrayLevelEmphasis
	−0.0139	wavelet-LLL_glszm_SmallAreaLowGrayLevelEmphasis

Note. Radiomics score = 2.0357 − 0.2915 × original_glszm_SmallAreaLowGrayLevelEmphasis + 0.3562 × exponential_gldm_DependenceNonUniformityNormalized + 0.0149 × exponential_glrlm_LongRunLowGrayLevelEmphasis − 0.2405 × gradient_glcm_InverseVariance + 0.2429 × gradient_glszm_SmallAreaLowGrayLevelEmphasis − 0.0717 × lbp-2D_glszm_SmallAreaEmphasis − 0.0717 × lbp-2D_glszm_SmallAreaHighGrayLevelEmphasis − 0.0717 × lbp-2D_glszm_SmallAreaLowGrayLevelEmphasis − 0.6683 × logarithm_gldm_DependenceNonUniformityNormalized + 0.3154 × logarithm_glszm_SmallAreaLowGrayLevelEmphasis + 0.2404 × logarithm_ngtdm_Busyness + 0.1940 × square_gldm_DependenceNonUniformityNormalized + 0.2003 × squareroot_gldm_DependenceNonUniformityNormalized − 0.0024 × squareroot_glszm_SmallAreaLowGrayLevelEmphasis + 0.1688 × wavelet-LLL_glrlm_LongRunLowGrayLevelEmphasis − 0.0139 × wavelet-LLL_glszm_SmallAreaLowGrayLevelEmphasis.

## Data Availability

The data used to prepare this manuscript are available within the manuscript and its Supplementary Materials. Additional information can be provided upon reasonable request to the corresponding author.

## Supplementary Materials

The following supporting information can be downloaded at: https://www.mdpi.com/article/10.3390/cancers18060949/s1, Supplementary Figure S1: The volume of PPAT and the Fibrosis Index; Supplementary Table S1: Correlation between PPAT fibrosis index and aggressiveness of prostate cancer; Supplementary Table S2: Correlation between PPAT fibrosis index and location of primary lesions in prostate cancer; Supplementary Table S3: Correlation between PPAT fibrosis and PSA levels, BMI, T staging and N staging.
