# Supplementary figures and images for: Fibrosis of Periprostatic Adipose Tissue: A Potential Marker of Prostate Cancer Aggressiveness
Source: Cancers (Basel). 2026 Mar 14;18(6):949. doi: 10.3390/cancers18060949 (PMC13024921; doi:10.3390/cancers18060949)

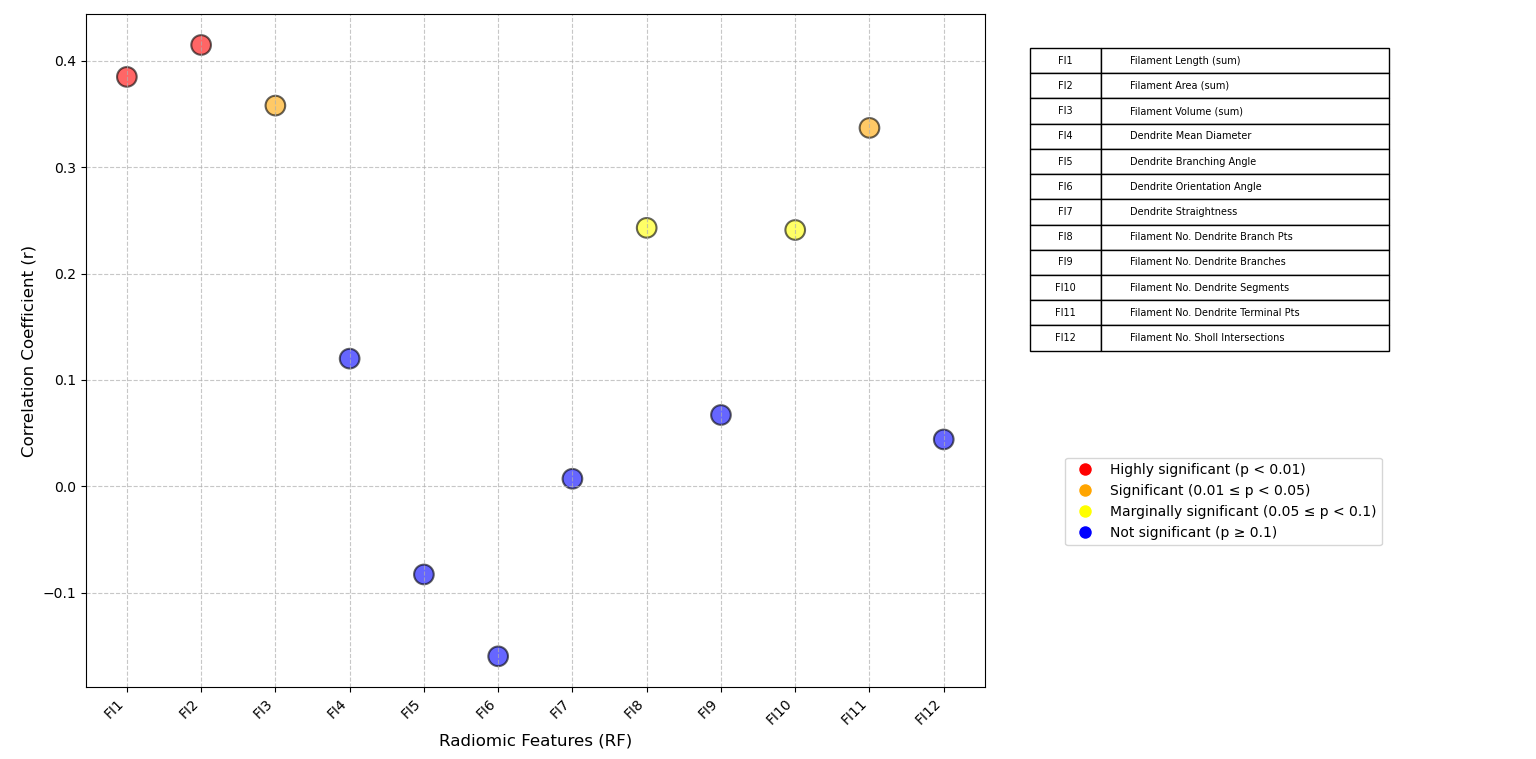

Supplement: Supplementary file 1 [file cancers-18-00949-s001.zip › Supplementary Figure S1-The volume of PPAT and the Fibrosis Index.png]
